# Predictive Factors for Functional and Anatomical Outcomes After Anti-VEGF Treatment for Macular Edema in Patients with Branch Retinal Vein Occlusion

**DOI:** 10.18502/jovr.v19i3.13531

**Published:** 2024-09-16

**Authors:** Catarina Cunha Ferreira, Ricardo Machado Soares, Joana Fernandes, Sofia Teixeira, Eduardo Saraiva, Lígia Ribeiro, Sofia Fonseca, Luís Silva, Filipe Sousa-Neves

**Affiliations:** ^1^Department of Ophthalmology, Unidade Local de Saúde Gaia/Espinho, Vila Nova de Gaia, Portugal; ^3^Catarina Cunha Ferreira: https://orcid.org/0000-0002-4356-9112

**Keywords:** Bevacizumab, Branch Retinal Vein Occlusion, Macular Edema, Optical Coherence Tomography, Prognostic Factors

## Abstract

**Purpose:**

This study aimed to identify predictive factors for the improvement of best-corrected visual acuity (BCVA) and reduction of central macular thickness (CMT) after treatment of macular edema (ME) due to branch retinal vein occlusion (BRVO) in a real-world setting.

**Methods:**

This retrospective study included patients with ME secondary to BRVO who were treated with intravitreal injection of bevacizumab as the first-line therapy and were followed up for 12 months. Demographic and clinical data, in addition to baseline spectral domain optical coherence tomography (SD-OCT) features, were considered as possible biomarkers of final BCVA and CMT. We also collected the data concerning the need for additional treatment including sectorial laser photocoagulation, change to another anti-VEGF agent, or intravitreal corticosteroid injection.

**Results:**

A total of 161 eyes were analyzed. BCVA significantly improved from baseline to 12-month follow-up (0.6 and 0.4 logMAR, respectively; *P* < 0.01). CMT decreased significantly during the follow-up period (from 498.0 to 325.0 μ m; *P* < 0.01). Final BCVA correlated positively with baseline BCVA (*P* < 0.01, *r* = 0.57). Older age at diagnosis and baseline SD-OCT findings including CMT, disruption of the retinal inner layers, retinal pigment epithelium (RPE) damage, and impairment of the ellipsoid zone and external limiting membrane negatively affected final BCVA (*P* < 0.01). Multiple regression analysis identified age and BCVA at baseline as the only independent predictors of final BCVA (*P *= 0.001 and *P* < 0.01, respectively). No association was found between clinical data, SD-OCT parameters, and final CMT.

**Conclusion:**

Various clinical and SD-OCT parameters are prognostically relevant for visual improvement in ME secondary to BRVO. Age at diagnosis and baseline BCVA were found to be independent predictors of visual outcome.

##  INTRODUCTION

Branch retinal vein occlusion (BRVO) is a common retinal vascular disease and macular edema (ME) is its primary complication and leading cause of visual impairment.^[1,2]^ BRVO leads to the blockage of venous drainage, resulting in hypoxia and upregulation of vascular endothelial growth factor (VEGF). ME likely results from increased vascular permeability and subsequent breakdown of the blood–retinal barrier following VEGF upregulation.^[3]^

Several treatment approaches have been developed for BRVO-associated ME, with anti-VEGF agents proving to be safe and effective in large randomized controlled clinical trials. These agents have gained approval from various regulatory authorities, establishing intravitreal anti-VEGF therapy as the predominant first-line treatment for ME secondary to BRVO.^[4,5,6,7,8]^

Nevertheless, treatment protocols used in controlled clinical trials can be significantly different from real-life clinical practice. Some patients may experience temporary or ineffective outcomes even after multiple intravitreal injections, leading to poor visual results.^[9]^ The possibility of using clinical and imaging features to predict visual and anatomical outcomes in patients with BRVO has raised recent interest.^[10,11,12]^

The purpose of this study is to identify predictive factors for the improvement of best-corrected visual acuity (BCVA) and reduction of central macular thickness (CMT) after anti-VEGF treatment in patients with ME due to BRVO in a real-world clinical setting.

##  METHODS

### Study Design and Setting

This is a retrospective, single-center, observational study. The recruited patients had been admitted between January 2017 and January 2022 with the diagnosis of ME secondary to BRVO and had been followed up for a minimum of 12 months. The study adhered to the tenets of the Declaration of Helsinki and met the criteria for exemption from ethics review according to the research policy at our center.

### Participants

The clinical records of consecutive patients with BRVO diagnosis were examined. The inclusion criteria were the presence of ME secondary to BRVO with a minimum of 12 months follow-up after treatment with intravitreal bevacizumab (Avastin, Genentech, California, USA) as first-line therapy. ME was defined as CMT ≥ 250 μ m and the presence of intraretinal or subretinal fluid (SRF) on spectral domain optical coherence tomography (SD-OCT). On the other hand, we excluded eyes with any concomitant ocular disease that could cause ME or reduce visual acuity (VA). This criterion included a history of the following conditions: vitreoretinal disease, age-related macular degeneration, diabetic retinopathy, uveitis, glaucoma, intraocular surgery in the previous three months, laser photocoagulation, previous intravitreal anti-VEGF or corticosteroid injection, and media opacities that compromised the quality of SD-OCT images.

All patients were treated with intravitreal bevacizumab (1.25 mg/0.05 mL) as first-line therapy. Patients received a loading dose of one intravitreal bevacizumab injection every four weeks for three months, followed by a *pro re nata* (PRN) regimen. Further treatment was administered if ME was present in SD-OCT and/or BCVA decreased ≥ 1 line in the Snellen chart.

### Data Collection

The collected baseline data included age at diagnosis, gender, comorbidities (high blood pressure, dyslipidemia, diabetes mellitus, and blood dyscrasia), glaucoma history, BRVO location (temporal superior, temporal inferior, superior hemiretinal, inferior hemiretinal, and macular), disease duration, BCVA, and lens status. BCVA was evaluated using the Snellen chart and the results were expressed in logMAR.^[13]^

At the 6- and 12-month follow-ups, we recorded the data on BCVA, the total number of injections, and further treatment during follow-up, including laser photocoagulation, intravitreal corticosteroid injection, switch of an anti-VEGF agent, or cataract surgery. SD-OCT images of the macula were obtained using the Spectralis OCT (Heidelberg Engineering, Heidelberg, Germany). CMT ( μ m) was defined as the retinal thickness within the central circle of the ETDRS (Early Treatment Diabetic Retinopathy Study) grid, which had a 1 mm diameter centered over the fovea, and was automatically calculated using the Spectralis. CMT was measured at baseline as well as at the 6- and 12-month follow-ups. Additional SD-OCT parameters analyzed at baseline included: intraretinal fluid (IRF); SRF; retinal hyperreflective foci (HF); disorganization of inner retinal layers (DRIL); epiretinal membrane (ERM); and integrity of the ellipsoid zone (EZ), external limiting membrane (ELM), and RPE. The DRIL disorganization was defined as the inability to recognize the boundaries between the outer plexiform layer and the inner nuclear layer, and/or the inner nuclear layer and the inner plexiform layer–ganglion cell layer complex in the central 1 mm foveal zone. All of the aforementioned parameters were assessed in terms of the absence or presence of the specific feature in question. The only exception was the last parameter, which was described as visible, partially visible without foveal involvement, partially visible with foveal involvement, or invisible.

Each SD-OCT scan was evaluated by two independent observers (C.C.F. and R.M.S.). Data with discrepancies were reanalyzed by a senior author (F.S.N.), and all investigators were masked to all clinical information during the SD-OCT assessments.

### Outcome Measures

The outcome measures of this study were the determination of BCVA and CMT at the 6- and 12-month follow-ups and the identification of demographic and tomographic factors predictive of final BCVA and CMT.

### Statistical Analysis

Statistical analysis was performed using the SPSS Statistics for Windows, version 26.0 (IBM Corp, Armonk, New York, United States). Continuous variables with a normal distribution were expressed as mean and SD, while those without a normal distribution were expressed as median and interquartile range (IQR). Categorical variables were described using absolute and relative frequencies. Univariate analysis was first performed using nonparametric tests, including the Mann–Whitney test and the Spearman correlation coefficient. A multiple regression model was used to identify independent predictive factors of final BCVA and CMT. Statistical significance was set at *P* < 0.05.

**Figure 1:**
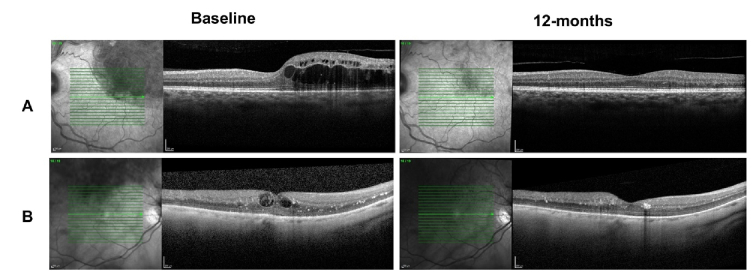
Macular OCT scans indicate a case (A) with BCVA of 0.7 logMAR, showing macular edema and integrity of the external retinal layers (top left), with good anatomical and functional response to therapy (BCVA of 0.1 logMAR at 12-month follow-up) (top right). The bottom scans represent a case (B) with the following characteristics: baseline BCVA of 1.0 logMAR, disorganization of the internal retinal layers, disruption of external limiting membrane and ellipsoid zone at the level of the fovea at baseline (bottom left), and insufficient functional response despite macular edema resolution after therapy (BCVA of 0.7 logMAR at 12-month follow-up) (bottom right).

**Table 1:** Clinical and demographic characteristics of patients enrolled in the study.


Age (yr, mean ± SD)		70.4 ± 10.5
Gender (female, *n* [ % ] )		71 (44.1%)
Disease duration (*n* [ % ] )		
	< 3 months	91 (56.5%)
	> 3 months	41 (25.5%)
	Unknown	29 (18.0%)
BRVO location (*n* [ % ] )		
	Temporal superior	73 (45.3%)
	Temporal inferior	50 (31.1%)
	Superior hemiretinal	12 (7.5%)
	Inferior hemiretinal	5 (3.1%)
	Macular branch	21 (13%)
Comorbidities (*n* [ % ] )		
	Hypertension	105 (65.2%)
	Dyslipidemia	51 (31.7%)
	Diabetes mellitus	39 (24.2%)
	Blood dyscrasia	3 (1.9%)
Glaucoma (*n* [ % ] )		21 (13%)
Phakic status (*n* [ % ] )		127 (78.9%)
	
	
BRVO, branch retinal vein occlusion; n, number of eyes; SD, standard deviation		

**Table 2:** Evolution of BCVA and CMT during the follow-up period.


**Parameter**	**BCVA (IQR), logMAR**	***P*** **-value**	**CMT (IQR), μ m**	***P*** **-value**
Baseline	0.6 (0.4–1.0)	–	490.0 (394.5–668.0)	–
6 months	0.4 (0.2–0.7)	< 0.01	326.5 (280.3–433.0)	< 0.01
12 months	0.4 (0.2–0.7)	< 0.01	325.0 (278.0–414.0)	< 0.01
	
	
Median (IQR). Differences were evaluated versus the initial visit BCVA, best corrected visual acuity; CMT, central macular thickness; IQR, interquartile range				

**Table 3:** BCVA and CMT evolution during the follow-up period according to the type of administered treatment.


**Treatment**	**Parameter**	**BCVA (IQR), logMAR**	***P*** **-value**	**CMT (IQR), μ m**	***P*** **-value**
Bevacizumab only	Baseline	0.5 (0.3–0.7)	< 0.01	468.0 (381.0–528.0)	< 0.01
	12 months	0.3 (0.2–0.5)	307.0 (278.0–408.2)	
Sectoral laser photocoagulation	Baseline	0.7 (0.4–1.4)	< 0.01	563.0 (403.0–709.5)	< 0.01
	12 months	0.5 (0.2–0.8)	331.0 (266.5–391.5)	
Switching to a 2nd anti-VEGF agent	Baseline	0.6 (0.4–1.5)	0.610	497.0 (366.0–650.0)	0.049
	12 months	0.6 (0.3–1.0)	384.0 (257.5–449.0)	
Corticosteroid injection	Baseline	0.9 (0.6–1.7)	0.009	656.5 (425.0–755.8)	0.004
	12 months	0.7 (0.4–1.0)	427.0 (366.0–650.0)	
	
	
Median (IQR) BCVA, best corrected visual acuity; CMT, central macular thickness; IQR, interquartile range; VEGF, vascular endothelium growth factor logMAR, logarithm minimum angle of resolution					

**Table 4:** SD-OCT parameters evaluated at baseline.


Central macular thickness ( μ m, median [IQR ] )	490.0 (394.5–668.0)
Intraretinal fluid (*n* [ % ] )	159 (98.8%)
Subretinal fluid (*n* [ % ] )	72 (44.7%)
Hyperreflective foci (*n* [ % ] )	105 (66.5%)
Disorganization of the retinal inner layers (*n* [ % ] )	109 (67.7%)
Disruption of ELM ${\;}^{a}$ (*n* [ % ] )	65 (40,4%)
Disruption of EZ ${\;}^{a}$ (*n* [ % ] )	71 (44.1%)
Disruption of RPE ${\;}^{a}$ (*n* [ % ] )	4 (2.5%)
Epiretinal membrane (*n* [ % ] )	10 (6.2%)
	
	
${\;}^{a}$ At the level of the fovea ELM, external limiting membrane; EZ, ellipsoid zone; RPE, retinal pigment epithelium; SD-OCT, spectral domain optical coherence tomography; IQR, interquartile range; n, number	

**Table 5:** Baseline predictive factors for visual and anatomical outcomes at 12 months.


**Parameter**		**Final BCVA**		**Final CMT**	
		**Median (IQR), logMAR**	**Univariate analysis (** ***P-*** **value)**	**Median (IQR), μ m**	**Univariate analysis (** ***P-*** **value)**
Gender	Female	0.4 (0.2–07)	0.596	316.0 (268.0–372.0)	0.172
	Male	0.4 (0.2–0.7)	327.5 (280.2–439.2)	
Disease duration	< 3 months	0.4 (0.2–0.8)	0.058	334.0 (269.0–414.0)	0.984
	> 3 months	0.5 (0.2–0.9)	328.0 (278.2–419.2)	
	Unknown	0.3 (0.1–0.5)	316.5 (282.0–432.2)	
BRVO location	TS	0.3 (0.2–0.7)	0.038	337.5 (282.0–434.8)	0.480
	TI	0.5 (0.2–0.8)	323.5 (257.8–369.2)	
	SHR	0.6 (0.3–1.7)	310.5 (264.5–360.0)	
	IHR	0.8 (0.6–1.8)	262.5 (208.2–499.8)	
	Macular	0.3 (0.2–0.5)	316 (273.0–420.0)	
Hypertension	Yes	0.4 (0.2—-0.7)	0.522	325.0 (281.0–416.0)	0.708
	No	0.4 (0.2—-0.8)	327.0 (264.0–412.2)	
Dyslipidemia	Yes	0.3 (0.2–0.7)	0.465	325.0 (280.0–444.0)	0.484
	No	0.4 (0.2–0.7)	324.5 (268.8–400.5)	
Diabetes mellitus	Yes	0.4 (0.3–0.7)	0.418	302.0 (271.0–449.0)	0.665
	No	0.3 (0.2–0.7)	329.0 (279.0–410.5)	
Blood dyscrasia	Yes	0.7 (0.2–0.7)	0.555	387.0 (233–387.0)	0.612
	No	0.4 (0.2–0.7)	324.0 (278.0–414.0)	
Glaucoma	Yes	0.5 (0.4–0.7)	0.257	320.5 (252.5–429.8)	0.605
	No	0.3 (0.2–0.7)	326.0 (280.0–407.00)	
Phakic status	Phakic	0.4 (0.2–0.7)	0.348	325.0 (280.0–414.0)	0.638
	Pseudophakic	0.4 (0.3–0.7)	328.0 (256.2–415.5)	
Subretinal fluid	Yes	0.5 (0.2–0.8)	0.086	321.0 (266.0–416.0)	0.490
	No	0.3 (0.2–0.6)	328.5 (282.5–414.0)	
DRIL	Yes	0.5 (0.2–0.8)	< 0.01	336.0 (279.2–436.8)	0.070
	No	0.3 (0.1–0.4)	308.0 (272.0–361.0)	
HF	Yes	0.4 (0.2–0.7)	0.401	310.0 (267.0–407.0)	0.101
	No	0.4 (0.2–0.9)	336.0 (292.0–421.5)	
ERM	Yes	0.4 (0.2–0.9)	0.667	363.0 (325.8–426.2)	0.060
	No	0.4 (0.2–0.7)	320.0 (273.0–410.5)	
RPE disruption ${\;}^{a}$	Yes	1.2 (0.6–1.9)	0.007	333.5 (230.8–345.5)	0.678
	No	0.4 (0.2–0.7)	323.0 (278.0–416.0)	
EZ disruption ${\;}^{a}$	Yes	0.7 (0.4–1.0)	< 0.01	338.0 (275.2–452.0)	0.393
	No	0.2 (0.1–0.4)	316.0 (278.0–381.0)	
ELM disruption ${\;}^{a}$	Yes	0.7 (0.4–1.0)	< 0.01	336.0 (269.0–438.5)	0.873
	No	0.3 (0.2–0.5)	321.5 (278.8–392.0)	
	
	
${\;}^{a}$ At the level of the fovea BCVA, best corrected visual acuity; BRVO, branch retinal vein occlusion; CMT, central macular thickness; DRIL, disorganization of the inner retinal layers; ELM, external limiting membrane; ERM, epiretinal membrane; EZ, ellipsoid zone; HF, hyperreflective foci; IHR, inferior hemiretinal; IQR, interquartile range; RPE, retinal pigment epithelium; SHR, superior hemiretinal; TI, temporal inferior; TS, temporal superior logMAR, logarithm minimum angle of resolution					

##  RESULTS

### Cohort Characterization 

A total of 161 eyes from 157 patients were included in the study. The demographic and clinical features of the sample are displayed in Table 1. The mean age at diagnosis was 70.4 ± 10.5 years and 44.1% of the patients were female. The most common BRVO location was superior temporal (*n* = 73, 43.5%), and the time from presentation to treatment was less than three months in 91 (56.5%) cases.

A mean of 3.2 ± 1.1 and 5.1 ± 2.0 injections per patient were administered at the 6- and 12-month follow-ups, respectively. Regarding additional therapy during follow-up, treatment for 8 (5%) eyes switched to another anti-VEGF agent and 24 (14.9%) eyes received intravitreal corticosteroid injections. Ninety-two (57.1%) eyes underwent sectoral scatter laser photocoagulation based on the findings of ischemia from the fluorescein angiography evaluation. It is also worth mentioning that seven (4.3%) eyes underwent cataract surgery.

### Best-corrected Visual Acuity (BCVA)

Considering the whole sample, BCVA improved significantly after treatment from 0.6 logMAR (IQR: 0.4–1.0) at baseline to 0.4 logMAR (IQR: 0.2–0.7) at both the 6- and 12-month follow-ups (*P* < 0.01 and *P* < 0.01, respectively) [Table 2]. Also, VA was maintained or improved in 130 (80.7%) eyes during the 12-month follow-up period. Sixty-four (39.8%) eyes had a BCVA improvement of three or more Snellen lines. Only nine (5.6%) eyes developed visual loss of three or more Snellen lines. No significant differences were found between the BCVA at the 6- and 12-month follow-ups (*P *= 0.854).

In eyes treated exclusively with bevacizumab, BCVA improved significantly from 0.5 logMAR (IQR: 0.3–0.7) at baseline to 0.3 logMAR (IQR: 0.2–0.5) at the 12-month follow-up (*P* < 0.01) [Table 3]. Concerning eyes receiving additional therapy during the follow-up period, BCVA improved from baseline to 12-month follow-up in both the laser photocoagulation group (0.7 logMAR [IQR: 0.4–1.4] and 0.5 logMAR [IQR: 0.2–0.8], respectively; *P* < 0.01) and the intravitreal corticosteroid group (0.9 logMAR [IQR: 0.6–1.7] and 0.7 logMAR [IQR: 0.4–1.0], respectively; *P *= 0.009) [Table 3]. Compared to eyes that did not receive additional treatment, eyes that received sectoral laser photocoagulation or intravitreal corticosteroid injections had a worse baseline BCVA (*P *= 0.003 and *P *= 0.003, respectively) and worse visual results after treatment (*P *= 0.017 and *P *= 0.001, respectively). In the group of eyes switching from bevacizumab to other anti-VEGF agents, no significant improvement in BCVA was noted at the 12-month follow-up compared to the baseline (0.6 logMAR [IQR: 0.3–1.0] and 0.6 [IQR: 0.4–1.5], respectively; *P *= 0.610) [Table 3]. Additionally, compared to the group of eyes that continued to receive the same anti-VEGF agent, the former group did not show a statistically significant difference at the baseline and final BCVA (*P *= 0.861 and *P *= 0.081, respectively).

### OCT Parameters

Table 4 presents the baseline SD-OCT findings. Considering the whole sample, we observed significant differences in the CMT between the baseline, 6-month follow-up, and 12-month follow-up [Table 2]. The median CMT decreased significantly from 490.0 μ m (IQR: 394.5–668.0) at baseline to 326.5 μ m (IQR: 280.3–433.0) at the 6-month follow-up (*P* < 0.01) and to 325.0 μ m (IQR: 278.0–414.0) at the 12-month follow-up (*P* < 0.01). No significant differences were found in terms of CMT between the 6 and 12 months of follow-up (*P *= 0.606).

Considering the group of eyes treated exclusively with bevacizumab, a significant decrease was noted in the median CMT from baseline to 12-month follow-up (468.0 μ m [IQR: 381.0–528.0] to 307.0 μ m [IQR: 278.0–408.2], respectively; *P* < 0.01) [Table 3]. Eyes receiving sectoral laser photocoagulation, intravitreal corticosteroid injection, or a switch to another anti-VEGF agent also showed a significant improvement in CMT during the follow-up period (*P* < 0.01, *P* = 0.004, and *P *= 0.049, respectively) [Table 3]. Compared to eyes that did not receive additional treatment, eyes that underwent sectoral laser photocoagulation had a greater baseline CMT (*P *= 0.007), but with no significant difference in final CMT (*P *= 0.697). In the group of eyes that received intravitreal corticosteroid injection, no statistically significant difference was found in the baseline CMT (*P *= 0.058) compared to eyes that received only bevacizumab injections; however, the final CMT results were worse in the corticosteroid group (*P* < 0.01). No significant differences were found in the baseline and final CMT in the group of eyes that switched to another anti-VEGF in comparison to those that did not receive additional therapies (*P *= 0.732 and *P *= 0.520, respectively).

### Univariate Analysis

The final BCVA correlated positively with baseline VA (*P* < 0.01, *r* = 0.57). In addition, a positive correlation was found between age at diagnosis and final BCVA (*P* < 0.01, *r* = 0.34). An association was noted between disease location and final BCVA, with the worst visual outcomes occurring in eyes with superior and inferior hemiretinal vein occlusion (HRVO) (*P *= 0.037). The final BCVA was negatively affected by an increased CMT (*P* < 0.01, *r* = 0.31), presence of DRIL (*P* < 0.01), as well as the disruption of RPE (*P *= 0.007), EZ (*P* < 0.01), and ELM (*P* < 0.01) [Figure 1; Table 5]. No association was found between the final CMT and the baseline clinical and tomographic parameters [Table 5].

### Multivariate Analysis

Multiple regression analysis was performed to identify independent predictive factors for the final BCVA. To this end, we incorporated baseline clinical and demographic parameters and OCT findings that significantly affected final BCVA in univariate analysis. Only baseline BCVA and age at diagnosis were demonstrated to be independent predictors of final BCVA in our series (*P *= 0.001 and *P* < 0.01, respectively). However, BRVO location (*P *= 0.579), baseline CMT (*P *= 0.132), presence of DRIL (*P *= 0.918), and disruption of RPE, EZ, and ELM (*P *= 0.414, *P *= 0.262, and *P *= 0.262, respectively) did not significantly predict the final BCVA in our sample.

Since none of the evaluated variables correlated significantly with the final CMT in univariate analysis, we did not conduct multiple regression analysis to identify independent predictors of final CMT.

##  DISCUSSION

In this study, we sought to evaluate the clinical and anatomical outcomes at 12-month follow-up in eyes with ME secondary BRVO treated with intravitreal bevacizumab as the first-line therapy. Additionally, we aimed to identify the demographic and tomographic factors that could predict final BCVA and CMT.

We observed a statistically significant improvement in BCVA from baseline to 12-month follow-up. Jaissle et al and Segal et al have reported similar gains in BCVA after bevacizumab therapy for ME secondary to BRVO, with improvements from 0.68 to 0.5 logMAR at one-year follow-up and 0.6 to 0.4 logMAR at 48-week follow-up, respectively.^[3,14]^ Additionally, 39.8% of eyes in our sample had a BCVA improvement of three or more Snellen lines. These findings are in line with previous prospective studies, demonstrating that similar results could be obtained in a real-world setting.^[15,16,17]^ Importantly, a number of previous randomized clinical controlled trials have presented greater VA gains after anti-VEGF therapy for BRVO.^[4,5,6,7]^ For instance, the VIBRANT trial identified a mean VA gain of 17 letters at the 24-week follow-up, and the BRAVO trial reported that 61.8% of patients showed an improvement of 15 letters or more at the one-year follow-up. These contrasting results could be attributed to variations in patient selection criteria, frequency of visits, and injection schedules between our study and the two mentioned trials.^[18]^

Consistent with prior studies, results of the current study suggest baseline BCVA as a significant predictor of visual outcomes, with better initial BCVA associated with greater improvement.^[11,15,19,23]^ We also observed a correlation between the age at diagnosis and the final BCVA, with older patients attaining worse visual outcomes. This association has been previously described in the literature.^[11,21,22,23]^ Interestingly, we found that eyes with HRVO had a poorer visual prognosis compared to eyes with BRVO. HRVOs are more extensive than BRVO and may represent a more serious condition. Nevertheless, our results contrast with those of the SCORE study, which reported that eyes with HRVO responded to treatment similarly to eyes that had BRVO in terms of VA changes.^[24]^

In our study, disease duration was not associated with final BCVA. Our findings differ from previous studies that consider the duration of disease from the onset of symptoms to the initial intravitreal injection as a prognostic factor for BCVA improvement.^[3,25]^ Notably, the disease duration was unknown in 18% of the patients in our sample. Given the retrospective nature of our study, missing information from the patient's medical records and memory bias may explain these results.

In accordance with previous literature, CMT decreased significantly in our sample from baseline to the 12-month follow-up.^[3,14][18][19]^ Costa et al reported that CMT declined steadily in the first six months of treatment and stabilized afterward.^[26]^ We observed a correlation between greater baseline CMT and poorer final BCVA, which is consistent with the findings reported by Jaissle et al and Segal et al.^[3,14]^

Regarding SD-OCT parameters, we observed the worst VA outcomes in eyes with DRIL and disruption of the RPE, EZ, and ELM. The presence of DRIL in macular OCT has been recently established as a biomarker for visual outcomes in BRVO.^[27,29]^ Mimouni et al reported that a change in the extent of DRIL disorganization after three monthly anti-VEGF injections for central retinal vein occlusion and BRVO was a predictor of VA improvement at the one-year follow-up.^[27]^ Additionally, Farinha et al showed that disruption of the external retinal layers negatively affected visual function.^[19]^ Similarly, Coscas et al found that visual prognosis depended on the presence of ELM and the integrity of the interface between the internal and external segments of photoreceptors.^[20]^

No association was found between the final BCVA and the presence of HF, SRF, and ERM. Segal et al also reported no significant correlation between visual outcomes and SRF extent or height and HF.^[14]^ Hoeh et al reported that SRF was not a predictor of functional or anatomical outcomes following treatment with bevacizumab in patients with BRVO.^[30]^ Likewise, Kang et al did not find a correlation between the number of HF at baseline and final BCVA.^[22]^

We did not find any association between demographic, clinical, and tomographic parameters and the final CMT in our study. Costa et al recently reported an association between ME recurrence and the presence of DRIL at baseline, but not the presence of HF or disruption of EZ,^[26]^ which is in line with previous studies.^[31,33]^ Moon et al confirmed that older age at diagnosis and longer duration of disease before treatment could be prognostic factors for ME recurrence at the two-year follow-up.^[31]^

Multiple regression analysis showed age and BCVA at baseline to be independent predictors of final VA in our sample. Likewise, Farinha et al introduced BCVA as an independent prognostic factor of final VA but also found an independent predictive role for RPE integrity.^[19]^ Similarly, using multiple regression analysis, Kang et al concluded that the strongest predictor of final BCVA was EZ integrity, followed by ELM integrity, and baseline BCVA.^[22]^ As mentioned, we did not notice an independent predictive value for SD-OCT measurements in relation to the final BCVA. Differences in study design, sample size, and criteria for the assessment of tomographic parameters could explain these diverging results, and further studies are needed to establish the role of SD-OCT measurements in BRVO prognosis.

The mean number of injections administered per patient at the 6- and 12-month follow-ups in our study is in line with those reported in the literature but is lower compared to those in randomized controlled trials.^[14,18,19]^

Important limitations are present in this study. Selection and information biases might have occurred due to the retrospective design we used. The follow-up period was relatively short, but BCVA and CMT at the six-month follow-up did not differ significantly from the final visit, showing that a six-month follow-up might be sufficient to predict long-term results. Another limitation of this study was that some patients received additional treatments on a PRN basis. These treatments included laser photocoagulation, intravitreal corticosteroid injections, and switching to a second anti-VEGF agent. This strategy could have affected the outcomes compared to other studies that used a different therapeutic approach.

In summary, although previous studies have sought predictive factors for successful treatment of ME due to BRVO, only a few of them have analyzed baseline clinical, demographic, and SD-OCT measurements to evaluate visual and anatomical outcomes after intravitreal bevacizumab injections as first-line therapy.^[14,22,28,33]^ According to our real-world findings, different clinical and SD-OCT parameters may be of prognostic relevance for visual improvement, including age at diagnosis, baseline BCVA, CMT, presence of DRIL, and disruption of RPE, EZ, and ELM. Specifically, age at diagnosis and baseline BCVA were found to be independent predictors of final BCVA.

## References

[B1] Jaulim A, Ahmed B, Khanam T, Chatziralli IP (2013). Branch retinal vein occlusion: Epidemiology, pathogenesis, risk factors, clinical features, diagnosis, and complications. An update of the literature Retina.

[B2] No authors listed (1984). Argon laser photocoagulation for macular edema in branch vein occlusion. The Branch Vein Occlusion Study Group Am J Ophthalmol.

[B3] Jaissle GB, Szurman P, Feltgen N, Spitzer B, Pielen A, Rehak M, et al (2011). ; Retinal Vein Occlusion Study Group. Predictive factors for functional improvement after intravitreal bevacizumab therapy for macular edema due to branch retinal vein occlusion Graefes Arch Clin Exp Ophthalmol.

[B4] Campochiaro PA, Heier JS, Feiner L, Gray S, Saroj N, Rundle AC, et al (2010). ; BRAVO Investigators. Ranibizumab for macular edema following branch retinal vein occlusion: Six-month primary end point results of a phase III study Ophthalmology.

[B5] Brown DM, Campochiaro PA, Singh RP, Li Z, Gray S, Saroj N, et al (2010). ; CRUISE Investigators. Ranibizumab for macular edema following central retinal vein occlusion: Six-month primary end point results of a phase III study Ophthalmology.

[B6] Campochiaro PA, Clark WL, Boyer DS, Heier JS, Brown DM, Vitti R, et al (2015). Intravitreal aflibercept for macular edema following branch retinal vein occlusion: The 24-week results of the VIBRANT study. Ophthalmology.

[B7] Clark WL, Boyer DS, Heier JS, Brown DM, Haller JA, Vitti R, et al (2016). Intravitreal aflibercept for macular edema following branch retinal vein occlusion: 52-week results of the VIBRANT study. Ophthalmology.

[B8] Heier JS, Campochiaro PA, Yau L, Li Z, Saroj N, Rubio RG, et al (2012). Ranibizumab for macular edema due to retinal vein occlusions: Long-term follow-up in the HORIZON trial. Ophthalmology.

[B9] Campochiaro PA, Sophie R, Pearlman J, Brown DM, Boyer DS, Heier JS, et al (2014). ; RETAIN Study Group. Long-term outcomes in patients with retinal vein occlusion treated with ranibizumab: The RETAIN study Ophthalmology.

[B10] Höh AE, Schaal KB, Dithmar S (2007). [Central and branch retinal vein occlusion. Current strategies for treatment in Germany, Austria and Switzerland] Ophthalmologe.

[B11] Kondo M, Kondo N, Ito Y, Kachi S, Kikuchi M, Yasuma TR, et al (2009). Intravitreal injection of bevacizumab for macular edema secondary to branch retinal vein occlusion: Results after 12 months and multiple regression analysis. Retina.

[B12] Ach T, Hoeh AE, Schaal KB, Scheuerle AF, Dithmar S (2010). Predictive factors for changes in macular edema in intravitreal bevacizumab therapy of retinal vein occlusion. Graefes Arch Clin Exp Ophthalmol.

[B13] Ferris FL 3rd, Kassoff A, Bresnick GH, Bailey I (1982). New visual acuity charts for clinical research. Am J Ophthalmol.

[B14] Segal O, Yavnieli R, Mimouni M, Rabina G, Geffen N, Moisseiev E, et al (2022). Optical coherence tomography biomarkers predicting visual acuity change after intravitreal bevacizumab injections for macular edema secondary to branch retinal vein occlusion. Ophthalmologica.

[B15] Jaissle GB, Leitritz M, Gelisken F, Ziemssen F, Bartz-Schmidt KU, Szurman P (2009). One-year results after intravitreal bevacizumab therapy for macular edema secondary to branch retinal vein occlusion. Graefes Arch Clin Exp Ophthalmol.

[B16] Kreutzer TC, Alge CS, Wolf AH, Kook D, Burger J, Strauss R, et al (2008). Intravitreal bevacizumab for the treatment of macular oedema secondary to branch retinal vein occlusion. Br J Ophthalmol.

[B17] Kriechbaum K, Michels S, Prager F, Georgopoulos M, Funk M, Geitzenauer W, et al (2008). Intravitreal Avastin for macular oedema secondary to retinal vein occlusion: A prospective study. Br J Ophthalmol.

[B18] Shalchi Z, Mahroo O, Bunce C, Mitry D (7). Anti-vascular endothelial growth factor for macular oedema secondary to branch retinal vein occlusion. Cochrane Database Syst Rev 2020;.

[B19] Farinha C, Marques JP, Almeida E, Baltar A, Santos AR, Melo P, et al (2015). Treatment of retinal vein occlusion with ranibizumab in clinical practice: Longer-term results and predictive factors of functional outcome. Ophthalmic Res.

[B20] Coscas G, Loewenstein A, Augustin A, Bandello F, Battaglia Parodi M, Lanzetta P, et al (2011). Management of retinal vein occlusion—Consensus document. Ophthalmologica.

[B21] Hasegawa T, Ueda T, Okamoto M, Ogata N (2014). Presence of foveal bulge in optical coherence tomographic images in eyes with macular edema associated with branch retinal vein occlusion. Am J Ophthalmol.

[B22] Kang HM, Chung EJ, Kim YM, Koh HJ (2013). Spectral-domain optical coherence tomography (SD-OCT) patterns and response to intravitreal bevacizumab therapy in macular edema associated with branch retinal vein occlusion. Graefes Arch Clin Exp Ophthalmol.

[B23] Ohashi H, Oh H, Nishiwaki H, Nonaka A, Takagi H (2004). Delayed absorption of macular edema accompanying serous retinal detachment after grid laser treatment in patients with branch retinal vein occlusion. Ophthalmology.

[B24] Scott IU, Vanveldhuisen PC, Oden NL, Ip MS, Domalpally A, Doft BH, et al (2012). ; SCORE Study Investigator Group. Baseline characteristics and response to treatment of participants with hemiretinal compared with branch retinal or central retinal vein occlusion in the standard care vs corticosteroid for retinal vein occlusion (SCORE) study: SCORE study report 14 Arch Ophthalmol.

[B25] Yoon YH, Kim HK, Yoon HS, Kang SW, Kim JG, Park KH, et al (2014). ; Korean RVO Study Group. Improved visual outcome with early treatment in macular edema secondary to retinal vein occlusions: 6-month results of a Korean RVO study Jpn J Ophthalmol.

[B26] Costa JV, Moura-Coelho N, Abreu AC, Neves P, Ornelas M, Furtado MJ (2021). Macular edema secondary to retinal vein occlusion in a real-life setting: A multicenter, nationwide, 3-year follow-up study. Graefes Arch Clin Exp Ophthalmol.

[B27] Mimouni M, Segev O, Dori D, Geffen N, Flores V, Segal O (2017). Disorganization of the retinal inner layers as a predictor of visual acuity in eyes with macular edema secondary to vein occlusion. Am J Ophthalmol.

[B28] Kang JW, Yoo R, Jo YH, Kim HC (2017). Correlation of microvascular structures on optical coherence tomography angiography with visual acuity in retinal vein occlusion. Retina.

[B29] Coscas F, Glacet-Bernard A, Miere A, Caillaux V, Uzzan J, Lupidi M, et al (2016). Optical coherence tomography angiography in retinal vein occlusion: Evaluation of superficial and deep capillary plexa. Am J Ophthalmol.

[B30] Hoeh AE, Ach T, Schaal KB, Scheuerle AF, Dithmar S (2009). Long-term follow-up of OCT-guided bevacizumab treatment of macular edema due to retinal vein occlusion. Graefes Arch Clin Exp Ophthalmol.

[B31] Moon BG, Cho AR, Kim YN, Kim JG (2018). Predictors of refractory macular edema after branch retinal vein occlusion following intravitreal bevacizumab. Retina.

[B32] Suzuki M, Nagai N, Minami S, Kurihara T, Kamoshita M, Sonobe H, et al (2020). Predicting recurrences of macular edema due to branch retinal vein occlusion during anti-vascular endothelial growth factor therapy. Graefes Arch Clin Exp Ophthalmol.

[B33] Arnarsson A, Stefánsson E (2000). Laser treatment and the mechanism of edema reduction in branch retinal vein occlusion. Invest Ophthalmol Vis Sci.

